# Phage treatment of an aortic graft infected with *Pseudomonas aeruginosa*

**DOI:** 10.1093/emph/eoy005

**Published:** 2018-03-08

**Authors:** Benjamin K Chan, Paul E Turner, Samuel Kim, Hamid R Mojibian, John A Elefteriades, Deepak Narayan

**Affiliations:** 1 Department of Ecology and Evolutionary Biology, Yale University, New Haven, CT, USA; 2 Program in Microbiology, Yale School of Medicine, New Haven, CT, USA; 3 Section of Plastic and Reconstructive Surgery, Department of Surgery, Yale School of Medicine, New Haven, CT, USA; 4 Department of Radiology and Biomedical Imaging, Yale School of Medicine, New Haven, CT, USA; 5 Section of Cardiac Surgery, Department of Surgery, Yale School of Medicine, New Haven, CT, USA

**Keywords:** phage therapy, prosthetic vascular graft infection, *Pseudomonas aeruginosa*, antibiotic resistance

## Abstract

Management of prosthetic vascular graft infections caused by *Pseudomonas aeruginosa* can be a significant challenge to clinicians. These infections often do not resolve with antibiotic therapy alone due to antibiotic resistance/tolerance by bacteria, poor ability of antibiotics to permeate/reduce biofilms and/or other factors. Bacteriophage OMKO1 binding to efflux pump proteins in *P. aeruginosa* was consistent with an evolutionary trade-off: wildtype bacteria were killed by phage whereas evolution of phage-resistance led to increased antibiotic sensitivity. However, phage clinical-use has not been demonstrated. Here, we present a case report detailing therapeutic application of phage OMKO1 to treat a chronic *P. aeruginosa* infection of an aortic Dacron graft with associated aorto-cutaneous fistula. Following a single application of phage OMKO1 and ceftazidime, the infection appeared to resolve with no signs of recurrence.

## INTRODUCTION

The widespread evolution of multi-drug-resistant (MDR) bacterial infections poses an escalating global threat to human health, highlighted by the recent World Health Organization’s first ever list of antibiotic resistant ‘priority pathogens’ [1]. Consequently, the increasing failure of traditional antibiotics has necessitated the development of novel antibacterial strategies, especially new therapies that target MDR bacteria which frequently colonize prosthetics associated with common surgeries. However, it is inevitable that pathogenic bacteria will evolve resistance against novel therapeutic intervention. Thus, new approaches ideally should target MDR bacterial pathogens while acknowledging that their further evolution would be unavoidable. In particular, these newly developed strategies could be designed to exert selection pressure on the bacteria to respond evolutionarily to therapy, by becoming on-average less biomedically problematic (e.g. reduced virulence, pathogenicity and/or transmissibility) [2–5]. This approach could be capable of successfully limiting MDR bacterial infections and additionally directing evolution of the pathogen in a clinically meaningful way.

Prosthetic vascular graft infections (PVGI) are catastrophic events which present serious challenges to surgeons and place heavy economic burdens on patients and the healthcare system. The reported incidence ranges from 0.6% to 9.5% depending on graft site [6, 7] and there are currently no readily identifiable or practical algorithms for their management. Traditional intervention strategies involve systemic antibiotics, debridement of infected tissue, partial-to-complete graft excision and secondary revascularization [8]. However, many patients presenting with vascular graft infections have significant comorbidities and are often critically ill, complicating surgical intervention and reducing likelihood of a positive clinical outcome. Despite best management, mortality and morbidity rates remain high with conservative estimates for both over 20% [9, 10]. Reinfection rates are also significant, highlighting the inadequacy of current treatment modalities at eradicating the infecting organism. As the number of procedures involving vascular grafts continues to increase with an aging population and prevalence of atherosclerosis and diabetes, new approaches to the management of PVGI are needed.

A common source of PVGIs is *Pseudomonas aeruginosa*, a ubiquitous Gram-negative, rod-shaped bacterium prevalent in natural and artificial environments [11] and listed as an increasingly antibiotic resistant priority pathogen [1]. Adaptation to myriad habitats has allowed *P. aeruginosa* to persist in many human-associated environments, most notably in hospitals, where it is increasingly associated with nosocomial infections [12]. These infections are difficult to manage, in part due to intrinsic antibiotic resistance resulting from decreased membrane permeability, active antibiotic efflux and other chromosomally encoded enzymes. Further complicating the problem of *P. aeruginosa* infections is their ability to form biofilms. Biofilm-mediated infections are notoriously difficult to manage, due to their generally greater resistance to chemical antimicrobials [13] and formation following sub-lethal concentrations of antibiotics [14]. Furthermore, slow-growing cells present in the biofilm (e.g. persister cells) may have sufficiently reduced metabolisms, resulting in resistance to bacteriostatic antibiotics that target metabolically active bacteria [15]. As a result, biofilms may also act as a reservoir for the dissemination of infections throughout the body which could greatly prolong infection duration and severity.

One potential alternative approach to using traditional antibiotics for prevention/treatment of bacterial infections is ‘phage therapy,’ the application of lytic bacteriophages (or ‘phages;’ viruses of bacteria) for the bio-control of bacteria. As one of the first classes of antimicrobials discovered in the modern era, their application has had a controversial past and their clinical use has not been fully accepted in Westernized countries. However, studies performed in the latter half of the 20th century [16], recent clinical trials [17, 18] and individual case reports [19] that demonstrate safety and potential efficacy have renewed interest in phage therapy as a possible mechanism by which antibiotic resistant and biofilm-associated infections might be controlled. As a class of antibacterials, phages are distinct from traditional chemical antibiotics in four seemingly beneficial ways: they are self-amplifying/limiting in the presence/absence of substrate (i.e. susceptible bacteria); they are often able to penetrate biofilms to reach infectious bacteria [20–22]; they are capable of infecting/killing persister cells; and their killing mechanism is distinct from those of traditional antibiotics. Exploiting the differences between antibiotics and phage therapy has been a driving force for continued research into the potential clinical utility of phage therapy.

**Figure 1.**
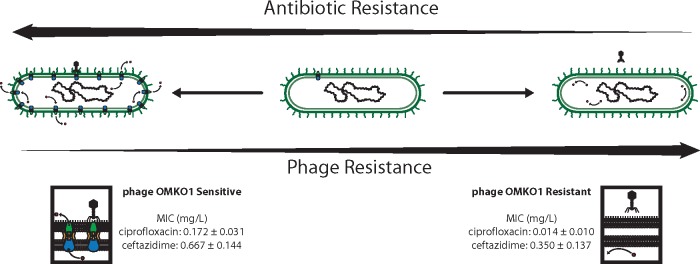
An ‘evolutionary-based strategy’ in phage therapy should be doubly effective. Top: if phage binding to surface-exposed proteins of efflux pumps causes lysis (killing) of infected cells, this simultaneously should exert selection pressure for bacteria to evolve phage resistance that concomitantly increases sensitivity to co-administered antibiotics due to inefficient efflux. Bottom: phage OMKO1 selects for increased sensitivity of MDR *P. aeruginosa* to antibiotics by forcing the desired trade-off [30]. Bacteria are either sensitive to the phage (and more resistant to antibiotics), left; or resistant to the phage (and more sensitive to antibiotics), right. Data previously reported in [30]

However, one obvious drawback to phage therapy is the clear evidence that bacteria can readily evolve resistance to phage infection [23, 24]. For example, phage attachment to a receptor binding-site exerts selection pressure for altered or down-regulated expression of the receptor, thereby allowing bacteria to escape phage infection [23]. Because evolved phage-resistance is virtually certain, modern approaches to phage therapy must acknowledge and capitalize on this inevitability. Trade-offs are often observed in biology, where organisms evolve one trait that improves fitness (a relative reproduction or survival advantage), while simultaneously suffering reduced performance in another trait [25–29]. Thus, phage therapy could be developed as an ‘evolutionary-based strategy’ that forces a trade-off of greatest relevance to the problem of widespread antibiotic resistance: utilize phages that drive MDR bacterial pathogens to evolve increased phage resistance by suffering increased sensitivity to chemical antibiotics (Fig. 1). This approach to phage therapy should be doubly effective; success is achieved when phage kills the target bacterium, and success is also achieved when bacteria evolve phage resistance because they suffer increased sensitivity to clinically approved antibiotics.

We recently identified phage OMKO1 that appears to utilize the outer membrane protein M of the mexAB- and mexXY-multidrug efflux systems of *P. aeruginosa* [30], exerting selection pressure for bacteria to evolve increased phage resistance that ‘trades-off’ with bacterial ability to maintain resistance to antibiotics (Fig. 1). These data suggest that bacteria evolve resistance to phage OMKO1 via mutations that alter efflux-pump proteins as binding sites for phage, thereby decreasing the ability for bacteria to extrude antibiotics and causing them to become antibiotic sensitive. This outcome is consistent with an evolutionary trade-off, which should allow phage OMKO1 to act synergistically with antibiotics in combination therapy. The use of this phage therapeutically, however, has not been previously tested. Numerous studies have examined the impact of phages on biofilms [21], but we are unaware of a case involving therapeutic application of phages to disrupt biofilms in a PVGI caused by MDR pathogen. Although methods to best leverage a phage-resistance/antibiotic-sensitivity trade-off in a clinical setting would be informed by consideration of dosage strategies (simultaneous vs alternating administration of phage and antibiotic); of pharmacokinetics and pharmacodynamics to optimize the trade-off; and of the possibility that MDR *P. aeruginosa* can simultaneously evolve resistance to phage and antibiotics, these questions are the targets of ongoing studies. Nevertheless, based on *in vitro* evidence that phage OMKO1 selects for re-sensitization to antibiotics in clinically relevant *P. aeruginosa* strains [30], we report the outcome of a single emergency case of phage therapy to treat a PVGI (chronic aortic Dacron graft) caused by *P. aeruginosa*.

## LABORATORY ASSAYS

**Figure 2.**
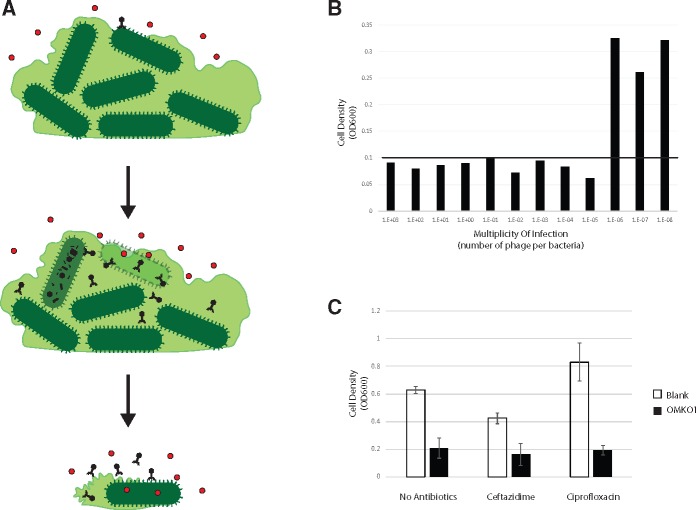
(**A**) Therapeutic concentrations of antibiotics are unable to penetrate biofilms due to poor permeability and depressed metabolism of biofilm constituents. Phage OMKO1, however, is able to replicate within bacteria present in biofilm. Next, biofilm instability occurs as phage OMKO1 replicates. Finally, with the biofilm disrupted, therapeutic concentrations of antibiotic can better reach target bacteria, and any cells resistant to phage OMKO1 infection are expected to be more susceptible to antibiotics (i.e., less capable of efflux). (**B**) Mean densities of bacteria previously grown for 72-h as biofilms on Dacron sections, following 24-h exposure to either ciprofloxacin or ceftazidime with and without phage OMKO1. (**C**) Mean densities of bacteria previously grown for 72-h as biofilms on Dacron sections, following 24-h exposure to differing amounts of phage OMKO1. The black horizontal line represents density equal to the reliable limit of detection (OD600 = 0.1). See text for details

Based on previous results [30], we hypothesized that additive effects, and potential synergy, between phage OMKO1 and ceftazidime could be utilized to disrupt *P. aeruginosa* biofilms when concentrations of antibiotic alone were too low to destroy the biofilm but sufficient to eliminate planktonic bacteria. Figure 2A illustrates this hypothetical synergy whereby phage-sensitive (antibiotic-resistant) cells in the biofilm are lysed, disrupting biofilm stability and exposing cells to lethal concentrations of antibiotic that kills phage-resistant (antibiotic-sensitive) bacteria. To examine potential synergy or additive effects and exclude concerns of phage/antibiotic antagonism prior to emergency application of phage OMKO1, we performed *in vitro* biofilm reduction assays using the strain isolated from fistular discharge of the patient.

Biofilms were grown on 3 mm × 3 mm sections of Dacron by inoculating each section in 150 µl 0.1 × LB broth in a 96-well dish with 50 µl of an overnight culture of *P. aeruginosa* isolated from fistular discharge of our patient. Dacron sections were removed from growth media after 72-h and rinsed with 200 µl of 0.1 × LB three times to remove planktonic cells. Following rinse, sections were added to 200 µl of LB medium containing treatment (phage OMKO1, ceftazidime or ciprofloxacin at 2 × MIC, antibiotic at 2 × MIC + phage OMKO1, or blank control). Following exposure to treatment for 24 h, Dacron sections were placed in fresh LB medium and allowed to incubate at 37°C for an additional 24 h without agitation. Dacron sections were then removed and cell density (optical density at absorbance wavelength 600 nm; OD_600_) was measured with an automated spectrophotometer (Tecan model Infinite F200 microplate-reader). Results (Fig. 2B) showed that phage OMKO1 combined with either ciprofloxacin or ceftazidime caused mean (*n* = 3) cell densities grown on Dacron sections to be statistically significantly lower than those exposed to either antibiotic alone (*t*-test with *P* < 0.001, ciprofloxacin; and *P* < 0.007, ceftazidime). Furthermore, treatment with phage alone was observed to significantly reduce mean cell densities relative to no treatment (*P* < 0.001). However, neither ciprofloxacin nor ceftazidime at 2 × MIC was sufficient to eliminate 72 h biofilms (*P* = 0.074, ciprofloxacin; *P* = 0.357, ceftazidime). These data indicated that the antibiotics were incapable of reducing cell densities in biofilms, whereas phage OMKO1 alone or in combination with antibiotic significantly reduced bacterial densities. The experiment also confirmed that phage OMKO1 and antibiotic showed no evidence of antagonistic interactions, suggesting that their co-administered treatment would not adversely affect biofilm reduction in the patient.

We then determined the minimum dose of phage OMKO1 required to remove biofilms in the absence of antibiotic to provide guidance for the *in vivo* efficacy, under the conservative assumption that possible phage-antibiotic synergy would not necessarily benefit the patient during treatment. To do so, we conducted replicated (*n* = 3) assays measuring minimum bactericidal titer, where differing concentrations of phage OMKO1 were used to attack biofilms initiated with constant cell densities identical to those used in the above-described biofilm reduction assays. Assay treatments consisted of serial 10-fold dilutions of phage OMKO1 starting at 10e10 plaque forming units (PFU) *per* ml. Cell densities (OD_600_) were then used to estimate the minimum multiplicity of infection (MOI: phage particles *per* cell) required to reduce biofilms on Dacron sections to a value less than OD_600_ = 0.1 (our limit of detection of viable bacterial cells using these methods). Results (Fig. 2C) showed that target biofilm reduction was achieved at MOI ≥ 0.00001, indicating that phage OMKO1 was highly effective at reducing biofilm densities even at very low relative frequencies of phage particles. The data suggested that a single treatment using 1,000 PFU of phage OMKO1 might be sufficient to effectively reduce a biofilm of ∼1e8 CFU of the *P. aeruginosa* patient strain, even if phage-antibiotic synergy *per se* could not be achieved during treatment.

## CASE PRESENTATION

**Figure 3.**
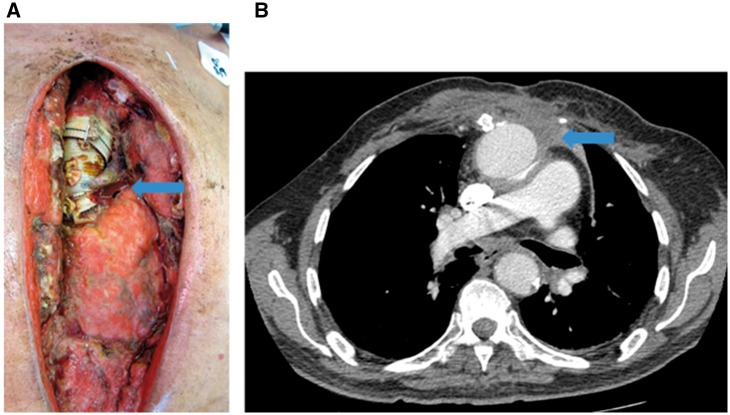
(**A**) Intraoperative photograph showing aortic graft and *P. aeruginosa* infection (arrow) over myocardium, taken during operation to debride and to wash out infected tissues. (**B**) Comparative CT image showing infected collection (arrow) and site of targeted aspiration during therapy

In July 2012, our 76-year old male patient underwent aortic arch replacement surgery with a Dacron graft for an aortic aneurysm. This was complicated by a *P. aeruginosa* mediastinal and aortic graft infection (confirmed by blood and deep wound culture) for which the patient returned to the operating room on multiple occasions for debridement and washout of the infected chest wall (Fig. 3A). The chest wall was eventually closed with omental and bilateral pectoralis major flaps. The patient was admitted again in early 2013 for recurrent *P. aeruginosa* infection (confirmed by blood and deep wound culture) and a new mediastinal fistula which drained purulent material. The patient was deemed too high risk for surgical replacement of his infected aortic graft after extensive discussions with his treating cardiac surgeon and multiple other consulting surgeons from other institutions. Therefore, the patient was conservatively treated with IV ceftazidime and superficial chest wall debridement. The patient was successfully discharged from the hospital, completed his multi-week course of intravenous ceftazidime (2 g IV q8h), and was switched to oral ciprofloxacin (750 mg q12h) as the isolated *P. aeruginosa* was susceptible to ciprofloxacin at this time. Over the course of the next year into 2014 the patient was admitted three more times for *P. aeruginosa* bacteremia (confirmed by cultures) which always began with subjective fevers and increased purulent drainage from his mediastinal fistula. CT imaging during the patient’s admission in October of 2013 showed increased perigraft fluid near the aortic root with surrounding phlegmonous changes in continuation with the mediastinal fistula tract (Fig. 3B). This poorly organized phlegmonous collection was thought to be the source of the mediastinal fistula discharge. However, the collection never became organized enough on subsequent CT scans to attempt drainage. During these admissions, his *P. aeruginosa* sample showed intermediate resistance to ciprofloxacin; therefore, the patient was treated with several week courses of intravenous ceftazidime that successfully suppressed the infection. Between each admission, attempts were made to restart oral ciprofloxacin after discussions with the infectious disease physician, but the patient eventually became septic after each attempt, necessitating intravenous ceftazidime treatment. The patient stayed abroad for over a year after his last hospital admission in May of 2014 where the patient continued to receive intravenous ceftazidime treatment. The patient presented to our clinic in late 2015 complaining of increased serosanguinous drainage from his mediastinal fistula. While the fistula had intermittently expressed mild serosanguinous drainage, the patient grew troubled because the fluid appeared to have become bloodier in nature. Based on this history, we became concerned that the perigraft, phlegmonous collection could be eroding into the aorta itself. The patient was not a candidate for elective surgical management and the patient, himself, wished to explore options other than indefinite antibacterial treatment. It was deemed at this time that the patient would make an ideal candidate for exploration of phage therapy with phage OMKO1.

## CASE MANAGEMENT AND OUTCOME

We proposed a procedure in which we would directly access the perigraft collection anterior to the aortic root by needle puncture using image guidance. At this site, we would distribute a mixture of phage OMKO1 and ceftazidime that had demonstrated synergism in a previous study [30]. This strategy was further supported by data in the current study (Fig. 2B and C) that used the strain isolated from the fistular discharge and confirmed re-sensitization to antibiotic in bacteria with evolved resistance to phage OMKO1. Importantly, these assays approximated the efficacy of *in vivo* biofilm breakdown, as it was unlikely that subsequent applications of phage OMKO1 would have been possible due to the complicated involvement of the proposed procedure.

### Purification and preparation of phage OMKO1

Application of phage OMKO1 required removal of endotoxins present in the phage lysate, because endotoxemia (and associated septic shock) was perceived as the greatest possible risk to the patient’s health. This endotoxin removal was accomplished *via* spin column (Pierce High Capacity Endotoxin Removal Spin Columns, Thermo Fisher) followed by dialysis in phosphate buffered saline. Limulus amebocyte lysate (LAL) testing was then performed by a third-party laboratory (Associates of Cape Cod, East Falmouth, MA) to determine endotoxin concentrations. Upon receiving endotoxin levels, dilution of the preparation was performed in injectable saline to produce a final concentration of 12.5 EU/ml and titer of 10e7 plaque forming units *per* ml (PFU/ml). This administered titer was determined to be acceptable and to likely exceed the minimum MOI capable of effectively reducing 72-h-old biofilms of the fistular-discharge strain in preliminary bactericidal titer assays (Fig. 2C), although the exact MOI at time of treatment was unknowable.

### Procedure

After the risks and benefits of the experimental procedure were discussed, the patient consented to the procedure. The Food and Drug Administration and Yale University Human Investigation Committee gave their approval for the use of phage OMKO1 as an emergency investigational new drug (FDA IND#16827). In January of 2016, pre-procedure CT imaging reconfirmed the perigraft, phlegmonous collection near the left-anterior aortic root which was first imaged in 2013. The collection had remained stable, but due to the chronicity of the infection and inflammation seen on imaging, heavy scarring was predicted to hinder our attempts at needle aspiration. The chest wall was punctured with a needle (away from the site of the mediastinal fistula) and advanced to the perigraft collection under direct CT guidance and in sterile fashion. Fluid could not be aspirated back or injected, confirming the heavy scarring in this area. Therefore, we withdrew the needle from the chest and applied 10 ml of phage OMKO1 (10e7 PFU/ml) and ceftazidime (0.2 g/ml) solution into the mediastinal fistula which was in continuity with the perigraft collection. A sterile dressing was placed over the fistula and the patient was admitted to a telemetry monitored bed for observation. The following day, the patient had no complaints, exhibited stable vital signs and had laboratory values within normal limits. We were confident that no injury to the aorta had occurred, and the patient was discharged still on his ceftazidime regimen. The patient returned to his home country shortly, thereafter.

Approximately 4 weeks post-procedure, the patient developed significant bleeding from the mediastinal wound. Due to serious concerns that an aortic perforation had occurred, the patient underwent emergency exploratory surgery. By operative report, aortic perforation was confirmed under direct visualization, caused by severe adhesions between the aorta and ectopic bone from remnants of the sternum. The patient underwent a partial (3 × 7 cm) aorta and graft excision and replacement, and repair of the mediastinal fistula. The entire graft could not be removed despite attempts to do so. Cultures taken during this time only revealed growth of *Candida* from the superficial chest wound which was appropriately treated. Ceftazidime was discontinued shortly after the surgery, and the patient has not had any evidence of recurrent infection despite remaining off of antibiotics (based on blood cultures, clinical symptoms and CT imaging).

## DISCUSSION

The argument for use of phage therapy in biofilm-associated infections is old and has a storied past. However, phage ability to disrupt biofilms where traditional antibiotics fail is a significant advantage (Fig. 2A) and should be considered for infections refractory to standard management practices. Phage such as OMKO1 that appear to force a clinically relevant trade-off may present an effective solution to the inevitable evolution of resistance by pathogenic bacteria. We verified the ability of phage OMKO1 to re-sensitize bacteria to antibiotics *in vitro* [14] prior to application, which provided valuable insight into the potential clinical outcome. This prior work provided preliminary data that suggested synergy could occur between phage OMKO1 and chemical antibiotics. Interestingly, the current study demonstrates the phage alone is capable of reducing bacterial biofilm densities in the presence/absence of antibiotic, suggesting phage-antibiotic antagonism should not be a concern during treatment against *P. aeruginosa* biofilms. Future work will further examine the conditions in which phage OMKO1 is effective alone versus in synergy with antibiotics in killing bacterial pathogens.

The long history of phage therapy in the former Soviet Union suggests that few, if any, side effects are associated with therapeutic application of bacteriophages [17, 18, 31, 32], and we did not anticipate any negative effects following use of phage OMKO1. In this case study, we did not observe any noticeable side effects associated with phage OMKO1. It appears to have been effective at biofilm reduction on prosthetic graft material, contributing to eradicating the *P. aeruginosa* infection. The standard of care for an infected thoracic aortic graft is complete removal of the infected graft, debridement of the surrounding infected tissue, and graft replacement [33]. However, this is many times deemed too risky, leading to more conservative treatments and/or lifelong suppressive antibiotic therapy [34]. Despite treatment, mortality rates for thoracic aortic graft infection range between 25% and 75% [34], and there is an approximately 20% risk of reinfection if the patient survives graft replacement [35]. In our case, only partial removal of the aortic graft was possible, greatly increasing the reinfection risk and necessitating continued lifelong suppressive antibiotics. Yet, the patient has not had a recurrent infection in the 18 months since discontinuing antibiotics after the emergent surgery. Due to these circumstances, we argue that the phage therapy played a significant role in contributing to the eradication of the *P. aeruginosa* infection. Eventual controlled trials examining phage application as adjunctives may reveal improved clinical outcomes in cases of recalcitrant infection, and we hope that exploratory studies such as this one can provide preliminary evidence suggesting that phage OMKO1 can greatly improve the effects of antibiotics for the removal of *P. aeruginosa* biofilms in PVGI.

Both the current case study and a recently reported treatment of an *Acinetobacter baumanii* infection highlight why the understanding of evolutionary biology is vital to the development and administration of phage therapy in humans. Schooley et al. reported the personalized phage-based therapy in a 68-year-old diabetic patient with necrotizing pancreatitis complicated by a MDR *A. baumanii* infection [19]. Nine different phages with lytic activity on the patient strain were identified, and were administered intravenously and percutaneously into abscess cavities using combinations (cocktails) of phages. However, the rise of phage resistance in the infecting bacterial population necessitated an iterative process of phage cocktail formulation, because of recurrent failure [19]. Although the patient’s infection ultimately resolved, this evolutionary process demonstrates one obvious challenge of phage therapy, where evolution of phage resistance occurs after treatment is administered [23, 24]. In contrast, the current case study indicates the fortuitous possibility for a single phage to apparently resolve the bacterial infection, where pre-treatment understanding of the evolutionary mechanism (phage-resistance/antibiotic-sensitivity trade-off) underlying bacterial resistance informed the choice of phage used in experimental therapy. Together, the two studies illustrate that improved understanding of both pre- and post-treatment mechanisms of phage resistance are crucial for further development of phage therapy strategies, for clinical medicine to capitalize on the general utility of phage therapy as an alternative to traditional antibiotics.
